# The association between access to key household resources and violence against women

**DOI:** 10.1038/s41598-023-37879-3

**Published:** 2023-07-28

**Authors:** Siddhartha Bandyopadhyay, Sanjukta Sarkar, Rudra Sensarma

**Affiliations:** 1 University of Birmingham, Birmingham, UK; 2 All India Institute of Medical Sciences, New Delhi, India; 3 Indian Institute of Management Kozhikode, Kozhikode, India

**Keywords:** Environmental economics, Socioeconomic scenarios

## Abstract

We provide the first comprehensive analysis of the association between two key household resources (drinking water and toilet access) and both non-partner violence (NPV) and intimate partner violence (IPV) experienced by women. We use data from a nationally representative household survey for India obtained from the latest (fourth) round of the National Family Health Survey conducted in 2015–16. We employ logistic regression method and also use inverse-probability-weighted regression adjustment to control for selection bias. We find that NPV decreases with access to drinking water, while IPV decreases with provision of toilets. These results are found to be robust to an alternative method viz. propensity score matching and selection on unobservables using the Rosenbaum bounds approach.

## Introduction

In most low-income countries, there is insufficient access to safe drinking water, adequate sanitation and hygiene facilities (WASH henceforth) at home^1^. This inadequacy particularly affects women and girls^2,3^. In addition to this being a public health problem, the literature indicates that lack of access to, or inadequate provision of WASH facilities can increase vulnerability to violence against women, VAW henceforth^4^. However, there is no comprehensive study that quantifies the association between these key household resources (WASH facilities) and VAW. We attempt to fill this gap by analyzing data from a large survey of Indian households to understand the association between WASH and VAW. In doing so, we analyze both IPV (intimate partner violence) and NPV (non-partner violence) and explain the different channels through which a lack of WASH facilities at home can lead to such violence.

While the lack of WASH can affect everyone, women and girls are disproportionately affected. In low-income countries, women and girls are frequently required to walk long distances in search of a water supply for drinking, cooking, laundry, as well as wait until dark to look for a private place to defecate and satisfy their sanitation needs. Stepping out of the house particularly at night exposes them to the risks of NPV in the form of physical and sexual violence^5,6^. Post-pubescent girls and women have the added difficulty of menstruation, which for a number of days per month increases their daily needs for water and sanitation^4^. Household water insecurity may increase the vulnerability of women to IPV as a penalty for failure to fulfil household activities dependent on water such as cooking and cleaning^7^.

The theoretical relationship between economic resources and violence is complex. In particular, the mechanism through which NPV and IPV are affected are different. For both types of violence, the simplest framework is to assume a ‘taste for violence’ which is nonetheless sensitive to factors that make it more costly to inflict violence. By taste for violence, we mean that the potential perpetrator enjoys inflicting violence i.e. it directly enters his utility function. This is as opposed to instrumental violence where the perpetrator does not directly enjoy violence but uses it as a tool to extract other goods or acts from which he gains utility. For example, in Bloch and Rao^8^, the perpetrator inflicts violence to extract dowry payments. For men who inflict NPV, one may hypothesise that they either have a direct taste for violence or use violence to extract sexual favours. For NPV, the access to WASH facilities lowers the expected gain for the opportunistic perpetrator as fewer women need to leave the house and away from other people. Applying the model by Becker^9^ that criminals weigh cost and benefits of committing crime, one can predict that the lowered opportunity to commit violence would make it costly for the potential perpetrator, as fewer women need to leave the house for their WASH needs. In line with the Becker framework, this would lower violence. While not the primary aim, improved WASH facilities lead to situational crime prevention i.e. it changes the environment causing a lowered opportunity for crime to occur^10,11^ thereby minimising expected reward via decreasing opportunities for perpetrators to inflict violence without risk of discovery, which is again consistent with the Becker framework.

For IPV, the relationship between economic resources and violence can be driven by changes in a women’s bargaining power. Access to WASH facilities frees up a woman’s time to pursue economic activities outside the house i.e. it leaves her time to supply wage labour which may increase her bargaining power within the household and also increase household income. More directly, the presence of WASH facilities may lead to a reduction in cognitive load and time pressure in their day, which may in turn reduce the opportunity for intra-household tensions that have the potential to lead to violent outcomes. The direction of causality could go in either direction, in that:

A) Better off household may experience fewer stressors than poorer households and experience reduced IPV, such households also have access to improved WASH;
B) A household that has easy accesses to WASH may experience reduced economic stress and decreased IPV.

Unfortunately, we cannot observe the direction of causality but both (1) and (2) are consistent with the observed associations.

A few studies have examined the link between resource availability and VAW. Cools and Kotsadam^12^ show that resource inequality (based on wealth, education and employment) is associated with higher intimate partner violence in Sub-Saharan Africa. Guimbeau et al.^13^ find that proximity to resources such as mineral deposits is associated with reduced acceptance of physical violence by women in India. Coming to household resources, there is very little work analyzing the relationship between toilet access and VAW. Gonsalves et al.^14^ quantify the association between toilet construction and reduced sexual violence in an urban township in South Africa using a mathematical simulation approach. There are also some qualitative studies on the link between lack of toilet facilities in households and perception of violence faced by women in India^15,16^. A few papers provide econometric analyses of the association between toilet availability and NPV^6,17,18^ but there is no existing evidence for IPV. Chaplin’s^19^ survey of the literature finds that the linkage between VAW and sanitation is poorly researched and documented. When it comes to the role of other key resources such as water, the literature is even more scant. The only study for the association between water access and VAW is a study on Nepal relating to IPV^7^.

Our study makes three distinct contributions to the literature. This is the first comprehensive study to analyze the association between the key household resources (water and sanitation) and both NPV and IPV. Second, our study uses data from a nationally representative survey which has greater potential for generalizability than local data-sets analyzed in some of the existing studies. We use household data for India obtained from the latest (fourth) round of the National Family Health Survey (NFHS-4 henceforth) conducted in 2015–16. Third, this is the only study to employ logistic regression and the inverse-probability-weighted regression adjustment to control for selection bias as well as examine the sensitivity of the results to selection on unobservables.

The NFHS is a large-scale, multi-round survey conducted on a representative sample of households throughout India. It is a nationally important source of data on population, health and nutrition indicators for each state and union territory (UT) and has been widely used in studies related to both IPV and NPV^6,17,18,20–25^. NFHS surveys are performed under the supervision of the Ministry of Health and Family Welfare (MoHFW), Government of India and the data collection is coordinated by the International Institute for Population Sciences (IIPS), Mumbai. Using data from NFHS-4^26^ provides us with a large sample to draw inferences from. The survey covers approximately 601,509 households from all 640 districts (across 29 states and 7 UTs) of India. Among those women who participated in the survey questions on IPV and NPV, we filter out the missing values to arrive at a final sample of 59,093 women for IPV and 76,580 for NPV. Second, NFHS-4 is distinct from the previous three rounds (conducted in 1992–93, 1998–99, 2005–06) as it provides district level estimates for the first time pertaining to a number of important socio-economic indicators.

We use logistic regression method to study the association between WASH and VAW, based on NFHS-4 data. However, we face the challenge of drawing causal inferences from a cross-sectional dataset. NFHS does not survey the same individuals across waves and therefore does not provide longitudinal information. While this is a limitation of the dataset, there is no other available dataset in India that has the kind of detailed information that NFHS provide. Moreover, being an observational dataset, the treatment variable representing individuals’ access to household WASH facilities is not random and may have possible relationships with both their observable and unobservable characteristics, causing selection bias. In the absence of appropriate instrumental variables, to mitigate a potential endogeneity problem, this study estimates treatment effects by applying a methodology that can control for the observed heterogeneity: inverse-probability-weighted regression adjustment (IPWRA henceforth) which can control for observed differences across the treatment and control groups. Individuals having access to household resources are considered to be the treatment group while non-users represent the counterfactual group or control group^27^. Robustness analysis is conducted using a standard matching method viz. Propensity Score Matching, PSM henceforth. Our review of the literature suggests that studies investigating the relationship between lack of sanitation resources in households and associated VAW have not used treatment effects estimation approaches. Thus, to the best of our knowledge, our study is the first to use such approaches (based on IPWRA) to mitigate selection bias in analyzing the link between lack of access to key household resources and VAW. However, due to the use of observational data, we are unable to fully control for the role of unobservables and hence we do not claim causal effects in our results.

The results of our logistic regression analysis show that improved access to drinking water has a statistically significant association with lower NPV experienced by women. The treatment effect obtained from the IPWRA method supports this result. We find using the IPWRA method that having access to drinking water reduces NPV by an average of 0.005. This reduction amounts to significant numbers of women being saved from NPV. For instance, the reduction in NPV amounts to a 10% decline or 371 women in our sample who would potentially experience lower NPV if they had access to drinking water. Projecting for the country, we are able to estimate that providing drinking water access can reduce NPV for 1.7 million women in India.

With respect to IPV, the logistic regression results show that improved access to toilets has a statistically significant association with reduction in IPV experienced by women. The IPWRA analysis produces similar results and suggests that access to toilets can reduce IPV for 1682 women in our sample and potentially 9.7 million in India. Thus, our results suggest that policy initiatives targeted at WASH and related behavioral change play a role in improving households’ welfare through the associated decrease in VAW. A challenge in this analysis is that IPRWA controls only for the role of observable factors. However, our results are robust to the presence of unobservable characteristics as found by sensitivity analysis conducted using the bounding approach^28^.

## Methods

For our empirical analysis, we use data from NFHS-4, 2015–2016. The Household Questionnaire lists all members who are usual residents of the household as well as visitors who have stayed the night before the interview. Basic demographic information on age, sex, marital status and schooling, pertaining to each person is collected. Information is also collected on characteristics of the dwelling unit such as source of drinking water, time to get to water source and the type of toilet facilities available. The information on age and sex of household members based on the household questionnaire is then used to further identify women who are eligible for individual interviews using the separate women’s questionnaire. Information on various background characteristics of women such as demographics, socio economic status, empowerment indicators and husband/ partner’s background are then collated through the women’s questionnaire^26^.

One woman (between the ages 15–49) per household is randomly selected in compliance with WHO guidelines on the ethical collection of such data in order to assess exposure to violence. To ensure that the violence subsample is nationally representative, special weights are then used to account for the random selection of only one woman per household. For the measurement of NPV, married and unmarried women are asked about their experience of physical as well as sexual violence committed by anyone, other than a current or most recent husband, in the last year. Additionally, information from currently married women about the violence committed by the current husband and from formerly married women about their most recent husband is collected to determine exposure to emotional, physical and sexual IPV^26^.

Our sample for assessing the relationship between WASH facilities and NPV consists of 76,580 currently, formerly and never married women and for our parallel analysis pertaining to IPV, we have a dataset of 59,093 currently and formerly married women. In both samples, only those women who are usual residents of their households have been considered. For IPV only currently and formerly married women are considered while for NPV, along with ever married women, never married women are also included. The percentage shares of women who experienced IPV and NPV, out of the women surveyed in each of the 29 states and 7 UTs of India are highlighted in Table 1. We observe that IPV is more prevalent than NPV everywhere and is the most reported (as a share of the women surveyed) in Bihar, Tamil Nadu and Manipur. NPV is the highest (as a share of the women surveyed) in Tamil Nadu, Telangana and Puducherry.

**Table 1:** Composition of Violence Against Women in States/UTs (in percent). Source: Authors’ calculations.

State/ UT	IPV	NPV
Andaman & Nicobar Islands	10.55	8.09
Andhra Pradesh	37.10	7.27
Arunachal Pradesh	27.30	3.12
Assam	20.58	3.53
Bihar	41.59	4.89
Chandigarh	14.49	1.11
Chhattisgarh	30.13	5.89
Dadra and Nagar Haveli	24.18	1.87
Daman and Diu	20.56	9.25
Delhi	25.30	4.59
Goa	12.20	2.06
Gujarat	17.54	2.53
Haryana	29.63	7.28
Himachal Pradesh	4.72	0.66
Jammu and Kashmir	10.88	2.55
Jharkhand	24.10	2.87
Karnataka	21.83	7.15
Kerala	12.68	1.35
Lakshadweep	5.26	
Madhya Pradesh	29.34	4.95
Maharashtra	18.57	2.65
Manipur	40.13	6.84
Meghalaya	25.83	7.84
Mizoram	14.21	1.51
Nagaland	12.58	1.86
Odisha	28.16	6.25
Puducherry	31.22	9.42
Punjab	18.13	4.19
Rajasthan	20.99	3.13
Sikkim	3.43	1.62
Tamil Nadu	40.86	12.84
Telangana	39.53	9.59
Tripura	23.39	2.41
Uttar Pradesh	29.28	5.20
Uttarakhand	9.69	3.21
West Bengal	23.82	3.81

The figures represent the number of women who reported experiencing any form of IPV (column 2) and NPV (column 3) as a percentage of the number of women surveyed in each State/ UT (Union Territory).

We begin our analysis by estimating a logistic regression where the dependent variable is the experience of violence by the respondent and the independent variable is access to water or toilets along with a host of socio-economic and socio-demographic variables. Next, we consider treatment effects analysis where access to WASH resources in a household represents a treatment wherein individuals using the facilities form the treatment group while non-users, i.e., those without access constitute the untreated group (counterfactual or control group). However, such assignment of the treatment is non-random which can lead to a potential selection bias in estimation of the treatment effects^29,30^. This is because the reasons for having access to WASH facilities can be based on observable household features of women as well as other unobservable characteristics, thus making the choice of usage endogenous. In order to mitigate this problem, our study employs treatment effects estimation using the IPWRA method^27^. The premise behind this method is to imitate randomization regarding the assignment of the treatment as is done in randomized controlled trials (RCTs)^31^. Linnemayr and Alderman^32^ point out that the external validity of RCTs is limited and recommend the use of matching estimators such as propensity score matching method to overcome the problems associated with RCTs.

Our objective is to measure the treatment effect (i.e., presence of WASH resources) on VAW. This is captured by the average treatment effect on the treated (ATET) computed as follows^33^:

$$ATET=E\left[{Y}_{i1}|{D}_{i}=1\right]-E\left[{Y}_{i0}|{D}_{i}=1\right],$$

where E[.] is the expectation operator, Y_i1_ is the potential outcome for the units that receive treatment (D = 1), Y_i0_ is the potential outcome for the units that do not receive treatment. The problem is that we do not observe the outcome of the treated units had they not received the treatment, i.e., E[Y_i0_|D_i_ = 1] but replacing these unobserved counterfactuals with the outcomes of the untreated i.e. E[Y_i0_|D_i_ = 0] may result in biased estimates^33^. Wooldridge^34^ suggests IPWRA as the way out where two models are estimated: the first model to predict treatment status (which gives us propensity scores) and the second model to predict outcomes (which uses propensity scores to calculate weights for the regression adjustment model). This means that only one model must be correctly specified for the regression coefficients to provide consistent average treatment effects. Thus, this procedure has been referred to as “doubly robust” in the sense that if one model is mis-specified, the other should still hold^34,35^.

In the first step of the IPWRA method, we estimate the treatment model using logistic regression with treatment status as the dependent variable and suitable covariates as explanatory variables. The predicted probabilities are known as propensity scores which can be expressed as: p(x) = Prob(D = 1|x) where x is the set of relevant pretreatment covariates. The second step is to fit weighted regression models of the outcomes for each treatment level and obtain the treatment-specific predicted outcomes, once again using logistic regression models. Each ‘treated’ person receives a weight equal to the inverse of the propensity score, and each ‘untreated’ person receives a weight equal to the inverse of one minus the propensity score. Finally, we compute the potential means of the treatment-specific predicted outcomes to obtain the average treatment effect on the treated (ATET). The IPWRA estimator is expressed as^33^:

$$ATET \left(IPWRA\right)={n}_{T}^{-1}{\sum }_{i=1}^{n}{D}_{i}\left[{r}_{T}^{*}\left(x,{\delta }_{T}^{*}\right)-{r}_{UT}^{*}\left(x,{\delta }_{UT}^{*}\right)\right],$$

where ${n}_{T}$ is the number of treated units out of the entire sample size of *n*,${r}_{T}^{*}\left(x,{\delta }_{T}^{*}\right)$ is the weighted regression model for treated (T) units with the inverse of $\widehat{p}\left(x\right)$ as the weight and ${r}_{UT}^{*}\left(x,{\delta }_{UT}^{*}\right)$ is the weighted regression model for untreated (UT) units with $1/(1-\widehat{p}\left(x\right))$ as the weight.

The use of the IPWRA method relies on two assumptions. The first is the conditional independence assumption (CIA) or unconfoundedness which means no unobservable variable affects both the likelihood of treatment as well as the outcome of interest after conditioning on covariates. We try to reduce this problem of selection on unobservables by following the Rosenbaum bounds approach^28^. The second assumption is the common support or overlap assumption which suggests that every observation comes with a positive probability of being both treated and controlled. We assess the overlap assumption by balancing on covariates. A covariate is said to be balanced when its distribution does not differ over treatment thresholds. We compute standardized differences which take into account both means and variances^36,37^. A perfectly balanced covariate has a standardized mean difference of zero and variance ratio of one^38^.

As a robustness check of our IPWRA results we also used the propensity score matching (PSM henceforth) method which depends on matching the individuals on their propensity scores and then comparing the outcomes to arrive at the ATET. Although we use PSM as a robustness check, we note that IPWRA has at least three advantages over PSM. The first one is the property of double robustness which makes it less prone to misspecification issues. The second advantage of IPWRA is the inclusion of controls for the observation’s baseline characteristics in the outcome model. Both IPWRA and PSM must satisfy the conditional independence assumption, which states that no unobservable variable affects both the likelihood of treatment and the outcome of interest after conditioning on covariates. Since IPWRA includes more covariates in the outcome model than PSM, which includes only the covariates in the treatment model, this assumption is more likely to hold with IPWRA than with PSM. The third improvement is that, unlike PSM, which compares each treatment observation to control observations that have a similar likelihood of being treated in a restrictive way, IPWRA implicitly compares every unit to every other unit while placing higher weights on observations that have a similar likelihood of being treated and lower weights on observations that are dissimilar^27^.

Now we discuss the choice of the variables in our analysis starting with the explanatory variables in the main logistic regression which include a treatment variable (drinking water or toilet facilities) along with the socio-economic and socio-demographic variables. Each of the treatment variables (drinking water and toilet facilities) is captured dichotomously where presence of the resource in the household is considered as the treatment or ${D}_{i}$=1 and absence as ${D}_{i}$=0. In line with Howard et al.^39^, we define drinking water variable as yes (or equals 1) if a household reports that it has water available on premises. If the household reports time taken for water collection (going and returning in minutes), we define it as no (or equals 0). Following Jadhav et al.^6^, we define toilet facility variable as yes (or equals 1) if a household reports that it has a facility available (flush, pit latrine), if no facility/bush/field, the variable is defined as no (or equals 0). Following the literature (see for instance Jadhav et al.^6^), the explanatory variables include place of residence (urban, rural), whether the dwelling has electricity (yes, no), education, ethnicity, religion and and region of residence (Northeast India, East India, North India, Central India, West India and lastly South India which is used as the reference category). A list of all potential covariates is provided in Table 2 even though the final choice of variables depended on criteria such as covariate balancing in the IPWRA (which we discuss below) and we included the same variables, along with the treatment variables, as the determinants of VAW in the logistic regression analysis. Table 3 shows a break-up of the sample across different individual and household characteristics.

**Table 2:** List of Variables and their Categories used in the Study.

Variables	Categories
Exposure to violence	
IPV (physical, emotional or sexual, in last 12 months)	No, yes
NPV (sexual or physical, in last 12 months)	No, yes
Individual characteristics	
Age	Number of years (15–49 years)
Ethnicity	Scheduled caste, scheduled tribe, other backward classes, other ethnicity
Marital status	Currently married, formerly married, never married
Religion	Christian, Hindu, Muslim, Sikh, Other religions (Buddhist, Jain, Jewish, Parsi etc.)
Education	Number of years (0–20 years)
Work status	Not working, working
Woman's control over how to spend her own money	No, yes
Intergenerational IPV (woman's father beat her mother)	No, yes
Husband/partner’s characteristics	
Education	Number of years (0–20 years)
Work status	Not working, working
Drinks alcohol	No, yes
Union characteristics	
Number of unions	Once, more than once
Psychological control by husband/ partner (respondent afraid of husband/partner)	No, yes
IPV justified by woman (if she goes out without telling husband/partner, neglects children, argues with him, refuses to have sex, doesn’t cook food properly, is unfaithful, disrespects in-laws)	No, yes
Marital control exercised by husband/ partner (jealous if respondent talks with other men, accuses her of infidelity, doesn’t allow to meet female friends, tries to limit contact with family, insists on knowing whereabouts, doesn’t trust with money)	No, yes
Household characteristics	
Access to clean drinking water	No, yes
Access to toilet facility	No, yes
Electricity	No, yes
De jure region of residence^a^	North India, Central India, East India, Northeast India, West India, South India
De jure place of residence	Urban, rural

^a^North consists of Chandigarh, Delhi, Haryana, Himachal Pradesh, Jammu & Kashmir, Punjab, Rajasthan, and Uttarakhand. Central consists of Chhattisgarh, Madhya Pradesh, and Uttar Pradesh. East consists of Bihar, Jharkhand, Odisha, and West Bengal. Northeast consists of Arunachal Pradesh, Assam, Manipur, Meghalaya, Mizoram, Nagaland, Sikkim, and Tripura. West consists of Dadra & Nagar Haveli, Daman & Diu, Goa, Gujarat, and Maharashtra. South consists of Andaman & Nicobar Islands, Andhra Pradesh, Karnataka, Kerala, Lakshadweep, Puducherry, Tamil Nadu, and Telangana.

**Table 3:** Description of the Sample.

Variables	IPV (N = 59,093)			NPV (N = 76,580)		
Exposure to violence	Numbers	Percent	Mean (S.D.)	Numbers	Percent	Mean (S.D.)
IPV						
No	44,275	74.924				
Yes	14,818	25.076				
NPV						
No				72,940	95.247	
Yes				3,640	4.753	
Individual characteristics						
Age (S.D.)			33.13 (8.05)			30.89 (9.14)
Ethnicity						
Scheduled caste	10,609	17.953		13,481	17.604	
Scheduled tribe	10,352	17.518		14,304	18.679	
Other backward classes	14,752	24.964		19,500	25.464	
Other ethnicity	23,380	39.565		29,295	38.254	
Marital status						
Currently married	56,243	95.177		60,146	78.540	
Formerly married	2850	4.823		3171	4.141	
Never married				13,263	17.319	
Religion						
Christian	3829	6.480		5773	7.539	
Hindu	44,887	75.960		56,433	73.692	
Muslim	7586	12.837		10,565	13.796	
Sikh	1225	2.073		1607	2.098	
Other religions	1566	2.650		2202	2.875	
Education (S.D.)			5.98 (5.2)			6.60 (5.22)
Work status						
No	39,727	67.228				
Yes	19,366	32.772				
Control over how to spend her own money						
No	33,174	56.139				
Yes	25,919	43.861				
Intergenerational IPV (woman's father beat her mother)						
No	47,583	80.522				
Yes	11,510	19.478				
Husband/partner’s characteristics						
Work status						
Not working	2395	4.053				
Working	56,698	95.947				
Education (S.D.)			7.55 (4.99)			
Drinks alcohol						
No	40,579	68.670				
Yes	18,514	31.330				
Union characteristics						
Number of unions						
Once	57,937	98.044				
More than once	1156	1.956				
Psychological control by husband/partner						
No	12,811	21.679				
Yes	46,282	78.321				
IPV justified by woman						
No	29,637	50.153				
Yes	29,456	49.847				
Marital control exercised by husband/partner						
No	30,932	52.345				
Yes	28,161	47.655				
Household characteristics						
Access to clean drinking water						
No	20,418	34.552		26,302	34.346	
Yes	38,675	65.448		50,278	65.654	
Access to toilet facility						
No	22,387	37.884		28,056	36.636	
Yes	36,706	62.116		48,524	63.364	
Electricity						
No	6332	10.715		8075	10.545	
Yes	52,761	89.285		68,505	89.455	
De jure region of residence						
South India	9138	15.464		11,118	14.518	
Northeast India	6942	11.748		10,530	13.750	
East India	10,584	17.911		13,130	17.145	
North India	12,539	21.219		16,756	21.880	
Central India	13,652	23.103		17,475	22.819	
West India	6238	10.556		7571	9.886	
De jure place of residence						
Urban	17,743	30.026		23,214	30.313	
Rural	41,350	69.974		53,366	69.687	

All observations with non-response to any question were eliminated before arriving at the final samples. Hence the number of observations is the same for all variables (i.e. 76,580 for NPV and 59,093 for IPV samples).

For the dependent variables in the logistic regression and in the outcome models of the IPWRA, we consider IPV and NPV which are modeled dichotomously such that the presence of any type of IPV (physical, sexual or emotional) = 1, absence = 0 and any type of NPV (physical or sexual) = 1, absence = 0. Following the literature^6,20,40^, the common regressors for the outcome models which are expected to be risk factors for the experience of both IPV and NPV include the woman’s age (15–49 years), marital status, ethnicity (scheduled caste, scheduled tribe, other backward classes), education (0–20 years) and religion (Christian, Hindu, Muslim, Sikh, others). For IPV, in addition to the above, the following regressors are included in the outcome model as risk factors, viz. number of unions (once, more than once), employment status of the woman (working, not working), woman has control over how to spend her own money (yes, no), whether the woman is afraid of husband/partner i.e. psychological control (yes, no), woman accepts IPV (yes, no), marital control exercised by husband/partner (yes, no) and whether the woman’s father beat her mother, i.e. intergenerational IPV (yes, no), husband/partner’s employment status (working, not working), husband/partner’s education (0–20 years) and husband/partner drinks alcohol (yes, no). These variables are explained in details in Table 2. The treatment model follows the same specification as mentioned earlier.

## Results

### Logistic regression model results for the relationship between household resources and VAW

We first report the results of the logit regression analysis to estimate the relationship between VAW and WASH. Table 4 presents the logistic regression results for the cases of IPV. The results in Panel A show that, improved access to drinking water does not have a statistically significant association with IPV. Among the control variables, the woman’s characteristics (such as age, marital status, education, work status etc.), husband’s characteristics (such as education, alcohol), religion, ethnicity and locational dummies seem to be significant determinants of IPV. The results for toilet facilities (Panel B of Table 4) show that toilet access has a negative and statistically significant coefficient, even after controlling for a host of control variables. In other words, access to toilet is associated with a reduction in IPV experienced by women. The odds ratio suggests that provision of toilet facility is associated with lower odds of experiencing IPV by 0.894 times. With respect to NPV, the logistic regression results (shown in Table 5) suggest that in the case of drinking water (see Panel A), water access has a negative and statistically significant association with NPV. Therefore, access to water within the house appears to reduce the NPV experienced by women. The odds ratio implies that water access can reduce the odds of experiencing NPV by 0.925 times. We also observe that control variables such as the woman’s age, education, ethnicity, marital status and region are significant determinants of NPV. Finally, in the case of toilet facilities (see Panel B of Table 5), we find that though improved access to toilets does not have a statistically significant association with reduction in NPV at 5% level of significance but the relationship is significant at the 10% level. This result is similar to the finding of Srinivasan^17^. The odds ratio can be interpreted to mean that toilet access reduces the odds of a woman experiencing NPV by 0.908 times.

**Table 4:** Logistic regression Estimation for the Association between IPV and WASH.

Panel A: drinking water				
Variables	Coefficient	[95% Conf. interval]	Odds ratio	[95% Conf. interval]
Drinking water	0.015	− 0.032–0.062	1.015	0.968–1.064
Electricity	− 0.165	− 0.232–(− 0.098)	0.847	0.792–0.905
Age	− 0.009	− 0.012– (0.006)	0.990	0.987–0.993
Scheduled tribe	− 0.248	− 0.324– (− 0.172)	0.780	0.723–0.841
Other backward classes	− 0.077	− 0.136 – (− 0.019)	0.925	0.872–0.981
Other ethnicity	− 0.255	− 0.326 –(− 0.184)	0.774	0.721–0.831
Currently married	0.297	0.190–0.404	1.346	1.210–1.498
Hindu	0.071	− 0.034–0.178	1.074	0.965–1.195
Muslim	0.167	0.043–0.291	1.182	1.044–1.337
Sikh	0.001	− 0.199–0.202	1.001	0.819–1.224
Other religions	− 0.230	− 0.397– (− 0.063)	0.794	0.672–0.938
Education	− 0.031	− 0.037– (− 0.025)	0.968	0.963–0.974
Work status	0.225	0.178–0.271	1.252	1.194–1.311
Control over how to spend own money	− 0.043	− 0.087–0.001	0.957	0.916–1.001
Intergenerational IPV	0.969	0.920–1.018	2.636	2.510–2.769
Husband/partner’s work status	0.004	− 0.102–0.111	1.004	0.902–1.117
Husband/partner’s education	− 0.020	− 0.025– (− 0.014)	0.980	0.974–0.985
Husband/partner drinks alcohol	0.905	0.860–0.950	2.472	2.363–2.587
More than one union	0.308	0.165–0.450	1.361	1.180–1.569
Psychological control by husband/partner	0.832	0.771–0.894	2.303	2.162–2.445
IPV justified by woman	0.479	0.435–0.523	1.614	1.544–1.687
Marital control exercised by husband/partner	1.136	1.090–1.181	3.114	2.976–3.259
Northeast India	− 0.147	− 0.238– (− 0.055)	0.863	0.787–0.945
East India	− 0.162	− 0.237– (− 0.087)	0.849	0.788–0.916
North India	− 0.456	− 0.535– (− 0.376)	0.633	0.585–0.686
Central India	− 0.204	− 0.275– (− 0.133)	0.815	0.759–0.874
West India	− 0.218	− 0.309– (− 0.127)	0.803	0.733–0.879
Rural	− 0.052	− 0.103–0.001	0.948	0.901–0.998
Intercept	− 2.552	− 2.797– (− 2.307)	0.077	0.060–0.099
No. of obs	59,093			
Wald chi^2^	9563.79			
Prob > chi^2^	0.000			
Pseudo R-squared	0.1905			
Log likelihood	− 26,939.35			

Panel B: toilet facility				
Variables	Coefficient	[95% Conf. interval]	Odds ratio	[95% Conf. interval]
Toilet facility	− 0.094	− 0.148– (− 0.039)	0.910	0.861–0.961
Electricity	− 0.147	− 0.215– (− 0.080)	0.862	0.806–0.922
Age	− 0.008	− 0.011– (− 0.005)	0.991	0.988–0.994
Scheduled tribe	− 0.255	− 0.331– (− 0.180)	0.773	0.718–0.835
Other backward classes	− 0.072	− 0.131– (− 0.013)	0.930	0.877–0.986
Other ethnicity	0.243	− 0.314– (− 0.172)	0.783	0.729–0.841
Currently married	0.298	0.191–0.405	1.347	1.210–1.499
Hindu	0.058	− 0.047–0.165	1.060	0.953–1.180
Muslim	0.172	0.048–0.295	1.187	1.049–1.344
Sikh	0.012	− 0.188–0.213	1.012	0.828–1.237
Other religions	− 0.236	− 0.403– (− 0.069)	0.789	0.668–0.933
Education	− 0.030	− 0.036– (− 0.024)	0.970	0.964–0.976
Work status	0.217	0.171–0.264	1.243	1.186–1.302
Control over how to spend own money	− 0.040	− 0.084–0.004	0.960	0.918–1.004
Intergenerational IPV	0.968	0.919–1.017	2.634	2.508–2.767
Husband/partner’s work status	0.005	− 0.101–0.112	1.005	0.903–1.118
Husband/partner’s education	− 0.018	− 0.025– (− 0.013)	0.981	0.975–0.986
Husband/partner drinks alcohol	0.903	0.857–0.948	2.467	2.358–2.581
More than one union	0.307	0.165–0.450	1.360	1.179–1.568
Psychological control by husband/partner	0.832	0.770–0.893	2.298	2.161–2.444
IPV justified by woman	0.478	0.434–0.522	1.613	1.543–1.686
Marital control exercised by husband/partner	1.135	1.089–1.180	3.112	2.974–3.256
Northeast India	− 0.110	− 0.203–0.017	0.895	0.816–0.983
East India	− 0.173	− 0.248– (− 0.097)	0.840	0.779–0.906
North India	− 0.445	− 0.524– (− 0.365)	0.640	0.591–0.693
Central India	− 0.212	− 0.284– (− 0.141)	0.808	0.752–0.867
West India	− 0.216	− 0.306– (− 0.125)	0.805	0.735–0.881
Rural	− 0.078	− 0.130– (− 0.025)	0.924	0.877–0.97
Intercept	− 2.518	− 2.762– (− 2.274)	0.080	0.063–0.102
No. of obs	59,093			
Wald chi^2^	9573.04			
Prob > chi^2^	0.000			
Pseudo R-squared	0.1907			
Log likelihood	− 26,93.79			

The table shows the estimated logistic regression models for each type of household resource for the IPV sample. One category from each categorical variable is omitted to avoid multi-collinearity.

**Table 5:** Logistic regression Estimation for the Association between NPV and WASH.

Panel A: drinking water				
Variables	Coefficient	[95% Conf. interval]	Odds ratio	[95% Conf. interval]
Drinking water	− 0.078	− 0.153– (− 0.004)	0.924	0.857–0.995
Electricity	− 0.164	− 0.274– (− 0.053)	0.848	0.759–0.947
Age	− 0.015	− 0.020– (− 0.010)	0.984	0.979–0.989
Scheduled tribe	− 0.218	− 0.342– (− 0.094)	0.803	0.710–0.910
Other backward classes	− 0.108	− 0.199– (− 0.017)	0.897	0.819–0.982
Other ethnicity	− 0.229	− 0.342– (− 0.116)	0.794	0.709–0.890
Formerly married	0.048	− 0.136–0.234	1.049	0.872–1.263
Never married	0.905	0.809–1.002	2.473	2.245–2.725
Hindu	0.062	− 0.104–0.229	1.064	0.901–1.257
Muslim	− 0.331	− 0.526– (− 0.135)	0.718	0.590–0.873
Sikh	0.246	− 0.062–0.556	1.279	0.939–1.744
Other religions	− 0.183	− 0.460–0.094	0.832	0.630–1.098
Education	− 0.031	− 0.039– (− 0.023)	0.968	0.961–0.976
Northeast India	− 0.928	− 1.072– (− 0.785)	0.395	0.342–0.456
East India	− 0.795	− 0.907– (− 0.684)	0.451	0.403–0.504
North India	− 1.019	− 1.137– (− 0.901)	0.360	0.320–0.405
Central India	− 0.709	− 0.808– (− 0.610)	0.492	0.445–0.543
West India	− 1.233	− 1.389– (− 1.077)	0.291	0.249–0.340
Rural	− 0.045	− 0.123–0.032	0.955	0.883–1.033
Intercept	− 1.620	− 1.897– (− 1.343)	0.228	0.170–0.306
No. of obs	76,580			
Wald chi^2^	1334.18			
Prob > chi^2^	0.000			
Pseudo R-squared	0.0445			
Log likelihood	− 13,989.53			

Panel B: toilet facility				
Variables	Coefficient	[95% Conf. interval]	Odds ratio	[95% Conf. interval]
Toilet facility	− 0.078	− 0.165– (− 0.007)	0.924	0.847–1.007
Electricity	− 0.147	− 0.258– (− 0.036)	0.863	0.772–0.964
Age	− 0.015	− 0.020– (− 0.010)	0.984	0.979–0.989
Scheduled tribe	− 0.208	− 0.332– (− 0.085)	0.811	0.717–0.918
Other backward classes	− 0.106	− 0.198– (− 0.015)	0.898	0.820–0.984
Other ethnicity	− 0.223	0.337– (− 0.109)	0.799	0.713–0.896
Formerly married	0.051	− 0.134–0.236	1.052	0.874–1.266
Never married	0.906	0.809–1.002	2.474	2.246–2.726
Hindu	0.057	− 0.110–0.224	1.058	0.895–1.251
Muslim	− 0.331	− 0.527– (− 0.136)	0.717	0.590–0.872
Sikh	0.241	− 0.067–0.550	1.273	0.934–1.734
Other religions	− 0.182	− 0.460–0.094	0.832	0.631–1.098
Education	− 0.030	− 0.038– (− 0.022)	0.969	0.961–0.977
Northeast India	− 0.915	− 1.060– (− 0.770)	0.400	0.346–0.462
East India	− 0.802	− 0.914– (− 0.690)	0.448	0.400–0.501
North India	− 1.018	− 1.136– (− 0.900)	0.361	0.321–0.406
Central India	− 0.715	− 0.814– (− 0.615)	0.489	0.442–0.540
West India	− 1.242	− 1.398– (− 1.086)	0.288	0.246–0.337
Rural	− 0.053	− 0.133–0.026	0.948	0.875–1.027
Intercept	− 1.494	− 1.785– (− 1.204)	0.224	0.167–0.299
No. of obs	76,580			
Wald chi^2^	1334.32			
Prob > chi^2^	0.000			
Pseudo R-squared	0.0444			
Log likelihood	− 13,990.09			

The table shows the estimated logistic regression models for each type of household resource for the NPV sample. One category from each categorical variable is omitted to avoid multi-collinearity.

### IPWRA: treatment and outcome model results

Next, we move to the IPWRA analysis starting with the treatment models that are necessary for estimating the propensity scores for each of the treatment variables. Table 6 shows the logistic regression results for the two treatment variables viz. drinking water and toilet facility pertaining to the IPV sample. The results show that women with more education and having electricity supply in their houses have greater access to both resources. With respect to region of residence, religion and ethnicity, there are significant differences across various regions, religions and ethnicities in terms of access to the resources. Further, women belonging to rural areas have lower access to all three resources. The results are similar for the NPV sample as illustrated in Table 7.

**Table 6:** Treatment Models Estimated from Logit Regression – IPV (N = 59,093).

Covariates	Drinking water [95% Conf. interval]	Toilet facility [95% Conf. interval]
Electricity	0.015 [− 0.044–0.074]	1.508 [1.424–1.592]
Northeast India	1.061[0.975–1.147]	2.875 [2.745–3.004]
East India	− 0.098 [− 0.161– (− 0.034)]	− 0.511 [− 0.583– (− 0.439)]
North India	0.514 [0.448–0.579]	0.831 [0.756–0.905]
Central India	− 0.008 [− 0.069–0.052]	− 0.318 [− 0.386– (− 0.251)]
West India	0.745 [0.667–0.824]	0.169 [0.086–0.252]
Rural	− 0.895 [− 0.941– (− 0.848)]	− 1.734 [− 1.791– (− 1.678)]
Education	0.068 [0.064–0.072]	0.145 [0.140–0.149]
Scheduled tribe	− 0.798 [− 0.863– (− 0.733)]	− 0.350 [− 0.428 − (− 0.272)]
Other backward classes	0.355 [0.294–0.416]	0.975 [0.904 − 1.406]
Other ethnicity	0.268 [0.222–0.313]	0.374 [0.322–0.426]
Hindu	− 0.219 [− 0.314– (− 0.125)]	− 0.847 [− 0.985– (− 0.709)]
Muslim	0.339 [0.229–0.499]	0.135 [− 0.019–0.289]
Sikh	1.237 [1.006–1.468]	0.792 [0.519–1.065]
Other religions	− 0.185 [− 0.320– (− 0.050)]	− 0.187 [− 0.384–0.011]
Intercept	0.726 [0.596–0.855]	− 0.204 [− 0.382– (− 0.026)]
No. of obs	59,093	59,093
LR chi^2^	9269.84	27,661.77
Prob > chi^2^	0.000	0.000
Pseudo R-squared	0.1217	0.3528
Log likelihood	− 33,458.271	− 25,377.03

The table shows the estimated treatment models for each type of household resource for the IPV sample. Confidence Intervals are in square brackets next to the point estimates. One category from each categorical variable is omitted to avoid multi-collinearity.

**Table 7:** Treatment Models Estimated from Logit Regression – NPV (N = 76,580).

Covariates	Drinking water [95% CI]	Toilet facility [95% CI]
Electricity	0.034 [− 0.018–0.087]	1.547 [1.473–1.621]
Northeast India	1.034 [0.960–1.107]	2.867 [2.757–2.977]
East India	− 0.118 [− 0.175– (− 0.061)]	− 0.547 [− 0.612– (− 0.483)]
North India	0.500 [0.442–0.558]	0.751 [0.685–0.817]
Central India	− 0.009 [− 0.063–0.045	− 0.394 [− 0.454– (− 0.333)]
West India	0.773 [0.701–0.844]	0.188 [0.113–0.263]
Rural	− 0.908 [− 0.948– (− 0.867)]	− 1.764 [− 1.814– (− 1.714)]
Education	0.064 [0.060–0.067]	0.131 [0.127–0.135]
Scheduled Tribe	− 0.780 [− 0.838– (− 0.723)]	− 0.380 [− 0.449– (0.311)]
Other backward classes	0.363 [0.309–0.416]	0.949 [0.887–1.011]
Other ethnicity	0.268 [0.222–0.313]	0.374 [0.322–0.426]
Hindu	− 0.215 [− 0.294– (− 0.135)]	− 0.947 [− 1.066– (− 0.828)]
Muslim	0.313 [0.220–0.406]	0.055
Sikh	1.201 [1.003–1.398]	0.738 [0.498–0.979]
Other religions	− 0.121 [− 0.234– (− 0.009)]	− 0.328 [− 0.497– (− 0.160)]
Intercept	0.693 [0.580–0.805]	− 0.073 [− 0.229–0.083]
No. of obs	76,580	76,580
LR chi^2^	11,769.27	35,605.27
Prob > chi^2^	0.000	0.000
Pseudo R-squared	0.1195	0.3538
Log likelihood	− 43,379.46	− 32,509.738

The table shows the estimated treatment models for each type of household resource for the NPV sample. Confidence Intervals are in square brackets next to the point estimates. One category from each categorical variable is omitted to avoid multi-collinearity.

Tables 8, 9, 10 and 11 present the outcome model estimates for both categories of violence. While the results are mixed, our broad findings are that the following variables have significant association with the woman’s experience of violence: her age, ethnicity, education, marital and work status, husband’s education , intergenerational IPV (in the case of IPV), control over how to spend money, whether husband drinks alcohol, number of unions, religion and empowerment (measured by whether the woman is afraid of her husband, whether she justifies violence, whether marital control is exercised by husband).

**Table 8:** IPWRA outcome model logit regression for ‘untreated’ sample- IPV.

Variables	Drinking water [95% CI]	Toilet facility [95% CI]
Age	− 0.004 [− 0.011–0.002]	− 0.003 [− 0.018–0.012]
Scheduled tribe	− 0.286 [− 0.431– (− 0.141)]	− 0.239 [− 0.565–0.087]
Other backward classes	− 0.047 [− 0.171–0.077]	0.012 [− 0.189–0.213]
Other ethnicity	− 0.098	− 0.272 [− 0.531– (− 0.012)]
Currently married	0.142 [− 0.125–0.408]	0.266 [− 0.316–0.847]
Husband/partner’s work status	0.001 [− 0.252–0.255]	− 0.241 [− 0.747–0.264]
Control over how to spend own money	− 0.040 [− 0.147–0.066]	− 0.150 [− 0.331–0.031]
Education	− 0.021 [− 0.035– (− 0.007)]	− 0.037 [− 0.062– (− 0.013)]
Husband/partner’s education	− 0.013 [− 0.026–0.000]	0.014 [− 0.018–0.046]
Intergenerational IPV	0.977 [0.865–1.089]	0.850 [0.637–1.063]
Husband/partner drinks alcohol	0.908 [0.805–1.012]	0.810 [0.636–0.984]
Work status	0.402 [0.292–0.511]	0.215 [0.026–0.403]
More than one union	0.152 [− 0.193–0.498]	0.066 [− 0.375–0.507]
Hindu	− 0.035 [− 0.243–0.173]	0.089 [− 0.513–0.691]
Muslim	0.102 [− 0.170–0.374]	0.346 [− 0.372–1.063]
Sikh	− 0.734 [− 1.455– (− 0.012)]	− 0.215 [− 1.077–0.647]
Other religions	− 0.090 [− 0.505–0.325]	0.474 [− 0.315–1.263]
Psychological control by husband/partner	0.770 [0.621–0.918]	0.848 [0.615–1.081]
IPV justified by woman	0.611 [0.507–0.714]	0.513 [0.311–0.715]
Marital control exercised by husband/partner	1.041 [0.935–1.148]	1.134 [0.923–1.345]
Intercept	− 2.984 [− 3.501– (− 2.467)]	− 2.972 [− 4.111– (− 1.833)]

The table shows the estimated outcome models for each type of household resource for the ‘untreated’ IPV sample. Confidence Intervals are in square brackets next to the point estimates. One category from each categorical variable is omitted to avoid multi-collinearity.

**Table 9:** IPWRA outcome model logit regression for ‘treated’ sample-IPV.

Variables	Drinking water [95% CI]	Toilet facility [95% CI]
Age	− 0.009 [− 0.013– (− 0.005)]	− 0.007 [− 0.011– (− 0.004)]
Scheduled tribe	− 0.285 [− 0.397– (− 0.172)]	− 0.326 [− 0.447– (− 0.204)]
Other backward classes	− 0.056 [− 0.131–0.020]	0.022 [− 0.065–0.109]
Other ethnicity	− 0.343 [− 0.431–0.255]	− 0.236 [− 0.331– (− 0.142)]
Currently married	0.202 [0.062–0.341]	0.146 [0.004–0.288]
Husband/partner’s work status	0.010 [− 0.124–0.143]	− 0.038 [− 0.183–0.108]
Control over how to spend own money	− 0.076 [− 0.132– (− 0.019)]	− 0.041 [− 0.101–0.018]
Education	− 0.030 [− 0.037– (− 0.023)]	− 0.029 [− 0.036– (− 0.021)]
Husband/partner’s education	− 0.029 [− 0.036– (− 0.021)]	− 0.025 [− 0.033– (− 0.017)]
Intergenerational IPV	1.047 [0.983–1.111]	1.086 [1.019–1.152]
Husband/partner drinks alcohol	0.968 [0.910–1.026]	0.917 [0.856–0.979]
Work status	0.181 [0.120–0.243]	0.231 [0.166–0.296]
More than one union	0.439 [0.257–0.620]	0.388 [0.192–0.583]
Hindu	0.026 [− 0.105–0.157]	− 0.112 [− 0.235–0.011]
Muslim	0.103 [− 0.047–0.253]	− 0.039 [− 0.182–0.104]
Sikh	− 0.286 [− 0.504– (− 0.067)]	− 0.439 [− 0.656– (− 0.223)]
Other religions	− 0.211 [− 0.425–0.004]	− 0.293 [− 0.487– (− 0.099)]
Psychological control by husband/partner	0.794 [0.717–0.870]	0.811 [0.732–0.890]
IPV justified by woman	0.505 [0.450–0.560]	0.546 [0.487–0.605]
Marital control exercised by husband/partner	1.195 [1.138–1.252]	1.194 [1.135–1.253]
Intercept	− 2.768 [− 3.055– (− 2.481)]	− 2.780 [− 3.081– (− 2.480)]

The table shows the estimated outcome models for each type of household resource for the ‘treated’ IPV sample. Confidence Intervals are in square brackets next to the point estimates. One category from each categorical variable is omitted to avoid multi-collinearity.

**Table 10:** IPWRA outcome model logistic regression for ‘untreated’ sample-NPV.

Variables	Drinking water [95% CI]	Toilet facility [95% CI]
Age	− 0.016 [− 0.028– (− 0.004)]	− 0.019 [− 0.037– (− 0.002)]
Scheduled tribe	− 0.321 [− 0.572– (− 0.070)]	0.393 [0.014–0.771]
Other backward classes	− 0.133 [− 0.312–0.046]	0.006 [− 0.270–0.283]
Other ethnicity	− 0.074 [− 0.323–0.175]	− 0.243 [− 0.640–0.155]
Formerly married	0.020 [− 0.603–0.643]	− 0.136 [− 0.824–0.552]
Never married	0.856 [0.636–1.077]	0.887 [0.534–1.240]
Education	− 0.035 [− 0.055– (− 0.016)]	− 0.023 [− 0.051–0.005]
Hindu	0.157 [− 0.240–0.554]	− 0.073 [− 0.792–0.646]
Muslim	− 0.290 [− 0.770–0.191]	− 0.564 [− 1.396–0.268]
Sikh	0.265 [− 0.615–1.145]	− 2.871 [− 5.016– (− 0.726)]
Other religions	− 0.050 [− 0.733–0.632]	1.702 [0.389–3.105]
Intercept	− 1.567 [− 2.217– (− 0.917)]	− 1.303 [− 2.388– (− 0.219)]
Age (in years)	− 0.016 [− 0.028–(− 0.004)]	− 0.019 [− 0.037– (− 0.002)]
Ethnicity		
Scheduled tribe	− 0.321 [− 0.572–(− 0.070)]	0.393 [0.014–0.771]
Other backward castes	− 0.133 [− 0.312–0.046]	0.006 [− 0.270–0.283]
General	− 0.074 [− 0.323–0.175]	− 0.243 [− 0.640–0.155]
Marital status		
Formerly married	0.020 [− 0.603–0.643]	− 0.136 [− 0.824–0.552]
Never married	0.856 [0.636–1.077]	0.887 [0.534–1.240]
Education (in years)	− 0.035 [− 0.055–(− 0.016)]	− 0.023 [− 0.051–0.005]
Religion		
Hindu	0.157 [− 0.240–0.554]	− 0.073 [− 0.792–0.646]
Muslim	− 0.290 [− 0.770–0.191]	− 0.564 [− 1.396–0.268]
Sikh	0.265 [− 0.615–1.145]	− 2.871 [− 5.016-(− 0.726)]
Others	− 0.050 [− 0.733–0.632]	1.702 [0.389–3.105]
Intercept	− 1.567 [− 2.217–(− 0.917)]	− 1.303 [− 2.388–(− 0.219)]

The table shows the estimated outcome models for each type of household resource for the ‘untreated’ NPV sample. Confidence Intervals are in square brackets next to the point estimates. One category from each categorical variable is omitted to avoid multi-collinearity.

**Table 11:** IPWRA outcome model logistic regression for ‘treated’ sample-NPV.

Variables	Drinking water [95% CI]	Toilet facility [95% CI]
Age	− 0.018 [− 0.024– (− 0.012)]	− 0.022 [− 0.029– (− 0.016)]
Scheduled tribe	− 0.271 [− 0.461– (− 0.082)]	− 0.286 [− 0.488– (− 0.084)]
Other backward classes	− 0.067 [− 0.186–0.052]	− 0.099 [− 0.230–0.032]
Other ethnicity	− 0.261 [− 0.402– (− 0.121)]	− 0.217 [− 0.361– (− 0.073)]
Formerly married	0.231 [− 0.001–0.464]	0.234 [0.003–0.466]
Never married	0.869 [0.746–0.933]	0.736 [0.612–0.861]
Education	− 0.036 [− 0.045– (− 0.026)]	− 0.043 [− 0.053– (− 0.033)]
Hindu	− 0.007 [− 0.217–0.204]	0.060 [− 0.139–0.260]
Muslim	− 0.440 [− 0.681– (− 0.200)]	− 0.423 [− 0.653– (− 0.193)]
Sikh	0.099 [− 0.252–0.450]	0.252 [− 0.082–0.587]
Other religions	− 0.121 [− 0.465–0.223]	− 0.177 [− 0.497–0.143]
Intercept	− 1.628 [− 1.972– (− 1.284)]	− 1.420 [− 1.766– (− 1.075)]

The table shows the estimated outcome models for each type of household resource for the ‘treated’ NPV sample. Confidence Intervals are in square brackets next to the point estimates. One category from each categorical variable is omitted to avoid multi-collinearity.

Results of balance checks post treatment effects estimation are shown in Tables 12 and 13 respectively. They illustrate that although we find substantial differences on many unweighted covariates between treatment and control groups in the raw data, once we use matching and weighting techniques to balance the treatment and comparison groups, we obtain good balance on all covariates—all standardized differences are close to 0 and nearly all variance ratios are close to 1. Figure A1 in the Appendix shows that the propensity score is balanced across treatment and comparison groups as the range of common support shows that there is overlap of the distributions of propensity scores in the treatment and comparison groups. We find that the matched sample on the right-hand side in every case is in the form of one line, which is encouraging as this indicates that there are no large deviations. After matching/ weighting is applied, the common support is good, which leads us to infer that both groups are similar on average^41^.

**Table 12:** Balance Checks- IPV (N = 59,093).

Panel A—PSM				
Variables	Standardized differences		Variance ratio	
	Raw	Matched	Raw	Matched
Drinking water				
Electricity	0.216	0.049	0.590	0.857
Northeast India	0.148	− 0.075	1.452	0.875
East India	− 0.245	− 0.032	0.679	0.935
North India	0.205	0.043	1.367	1.053
Central India	− 0.197	0.010	0.789	1.017
West India	0.160	0.041	1.553	1.095
Rural	− 0.521	− 0.033	1.800	1.016
Education	0.509	0.051	1.332	1.101
Scheduled tribe	− 0.349	0.030	0.573	1.088
Other backward classes	0.087	− 0.011	1.040	0.996
Other ethnicity	0.339	− 0.006	1.574	0.996
Hindu	− 0.237	0.053	1.372	0.954
Muslim	0.225	− 0.051	1.739	0.922
Sikh	0.187	− 0.017	5.672	0.928
Other religions	− 0.035	− 0.020	0.817	0.885
Toilet facility				
Electricity	0.582	0.020	0.199	0.856
Northeast India	0.543	− 0.126	7.274	0.858
East India	− 0.450	0.015	0.487	1.048
North India	0.350	0.025	1.748	1.025
Central India	− 0.397	0.045	0.624	1.102
West India	0.057	− 0.034	1.160	0.927
Rural	− 0.817	− 0.082	2.868	1.028
Education	0.896	0.179	1.493	1.196
Scheduled tribe	− 0.155	0.028	0.773	1.063
Other backward classes	− 0.102	0.034	0.960	1.019
Other ethnicity	0.532	− 0.070	2.151	0.960
Hindu	− 0.525	0.148	2.208	0.922
Muslim	0.300	− 0.167	2.132	0.807
Sikh	0.208	0.028	7.760	1.140
Other religions	0.090	0.025	1.764	1.138

Panel B—IPWRA weighting				
Variables	Standardized differences		Variance ratio	
	Raw	Weighted	Raw	Weighted
Drinking water				
Electricity	0.216	0.054	0.590	0.858
Northeast India	0.148	− 0.106	1.452	0.814
East India	− 0.245	− 0.011	0.679	0.979
North India	0.205	0.035	1.367	1.045
Central India	− 0.197	0.043	0.789	1.068
West India	0.160	0.047	1.553	1.121
Rural	− 0.521	− 0.017	1.800	1.009
Education	0.509	0.044	1.332	1.153
Scheduled tribe	− 0.349	0.038	0.573	1.093
Other backward classes	0.087	− 0.006	1.040	0.998
Other ethnicity	0.339	− 0.017	1.574	0.985
Hindu	− 0.237	0.052	1.372	0.952
Muslim	0.225	− 0.059	1.739	0.898
Sikh	0.187	− 0.026	5.672	0.870
Other religions	− 0.035	− 0.011	0.817	0.937
Toilet facility				
Electricity	0.582	0.057	0.199	0.769
Northeast India	0.543	0.002	7.274	1.003
East India	− 0.450	− 0.023	0.487	0.947
North India	0.350	0.035	1.748	1.039
Central India	− 0.397	0.027	0.624	1.051
West India	0.057	− 0.029	1.160	0.934
Rural	− 0.817	− 0.130	2.868	1.058
Education	0.896	0.228	1.493	1.246
Scheduled tribe	− 0.155	0.046	0.773	1.098
Other backward classes	− 0.102	0.003	0.960	1.001
Other ethnicity	0.532	0.010	2.151	1.008
Hindu	− 0.525	0.047	2.208	0.966
Muslim	0.300	− 0.091	2.132	0.861
Sikh	0.208	0.011	7.760	1.064
Other religions	0.090	0.024	1.764	1.140

**Table 13:** Balance checks NPV (N = 76,580).

Panel A- PSM				
Variables	Standardized differences		Variance ratio	
	Raw	Matched	Raw	Matched
Drinking water				
Electricity	0.216	0.045	0.587	0.865
Northeast India	0.148	− 0.060	1.390	0.906
East India	− 0.253	− 0.022	0.659	0.951
North India	0.206	0.026	1.355	1.030
Central India	− 0.192	0.021	0.791	1.035
West India	0.159	0.037	1.583	1.090
Rural	− 0.519	− 0.018	1.779	1.008
Education	0.488	0.050	1.220	1.087
Scheduled tribe	− 0.333	0.036	0.609	1.096
Other backward classes	0.074	− 0.005	1.038	0.998
Other ethnicity	0.338	− 0.005	1.553	0.996
Hindu	− 0.237	0.017	1.326	0.986
Muslim	0.227	− 0.031	1.693	0.953
Sikh	0.184	0.003	5.392	1.017
Other religions	− 0.027	− 0.004	0.859	0.977
Toilet facility				
Electricity	0.576	0.030	0.206	0.801
Northeast India	0.591	− 0.129	7.010	0.878
East India	− 0.461	− 0.002	0.467	0.995
North India	0.338	0.078	1.688	1.083
Central India	− 0.417	0.043	0.608	1.101
West India	0.050	− 0.025	1.145	0.941
Rural	− 0.813	− 0.074	2.868	1.024
Education	0.837	0.148	1.269	1.133
Scheduled tribe	− 0.116	− 0.003	0.835	0.995
Other backward classes	− 0.129	0.008	0.944	1.005
Other ethnicity	0.521	0.013	2.086	1.008
Hindu	− 0.568	0.157	2.198	0.936
Muslim	0.315	− 0.099	2.142	0.879
Sikh	0.208	− 0.087	7.731	0.742
Other religions	0.095	− 0.039	1.773	0.846

Panel B- IPWRA weighting				
Variables	Standardized differences		Variance ratio	
	Raw	Weighted	Raw	Weighted
Drinking water				
Electricity	0.216	0.052	0.587	0.861
Northeast India	0.148	− 0.096	1.390	0.846
East India	− 0.253	− 0.007	0.659	0.986
North India	0.206	0.039	1.355	1.049
Central India	− 0.192	0.037	0.791	1.059
West India	0.159	0.038	1.583	1.101
Rural	− 0.519	− 0.014	1.779	1.007
Education	0.488	0.048	1.220	1.163
Scheduled tribe	− 0.333	0.042	0.609	1.096
Other backward classes	0.074	− 0.011	1.038	0.995
Other ethnicity	0.338	− 0.015	1.553	0.988
Hindu	− 0.237	0.049	1.326	0.960
Muslim	0.227	− 0.065	1.693	0.895
Sikh	0.184	− 0.011	5.392	0.939
Other religions	− 0.027	− 0.016	0.859	0.913
Toilet facility				
Electricity	0.576	0.060	0.206	0.761
Northeast India	0.591	− 0.034	7.010	0.953
East India	− 0.461	− 0.023	0.467	0.944
North India	0.338	0.055	1.688	1.064
Central India	− 0.417	0.041	0.608	1.082
West India	0.050	− 0.037	1.145	0.913
Rural	− 0.813	− 0.101	2.868	1.042
Education	0.837	0.189	1.269	1.193
Scheduled tribe	− 0.116	0.030	0.835	1.056
Other backward classes	− 0.129	− 0.003	0.944	0.998
Other ethnicity	0.521	0.017	2.086	1.013
Sikh	− 0.568	0.071	2.198	0.961
Other religions	0.315	− 0.078	2.142	0.884
Other backward classes	0.208	− 0.024	7.731	0.883
Other ethnicity	0.095	− 0.006	1.773	0.969

### IPWRA and PSM results for the relationship between household resources and IPV

Table 14 presents the treatment effects results for the association between access to WASH resources and VAW using IPWRA and PSM methods. We begin the discussion of our results with reference to the reduction in IPV achieved by each household resource starting with toilet availability. The estimates of the respective ATETs from the IPWRA analysis suggest that, having access to toilets is associated with reduced exposure to IPV by an average of 0.026 (and the ATET is statistically significant at the 5% level). Using the PSM method, the reduction turns out to be 0.044 (and statistically significant at the 1% level). To arrive at the estimated number of women who experience lower violence, we apply these percentage point reductions from IPWRA to the potential outcome means (POM) shown in Table 15 (the POMs are the mean outcomes of the untreated individuals). For instance, 22.9% of women without access to toilets experience IPV (as per Table 15) and when women have access to toilets this figure goes down by 0.026 to 20.3%. Based on the proportion of women who experience IPV, this is a reduction of 11%. For our sample of 59,093 women who answered the survey question on IPV out of whom 14,818 said they experienced IPV, this translates into 1682 women who could be saved from violence from their intimate partner if they are provided access to drinking water.

**Table 14:** Average Treatment Effects on the Treated (IPV N = 59,093 and NPV N = 76,580).

Outcomes	Drinking water		Toilet facility	
	IPWRA	PSM	IPWRA	PSM
IPV	− 0.006 [− 0.014–0.003]	− 0.035 [− 0.046– (− 0.025)]	− 0.026 [− 0.047– (− 0.005)]	− 0.045 [− 0.067– (− 0.022)]
NPV	− 0.005 [− 0.009– (− 0.001)]	− 0.006 [− 0.010– (− 0.002)]	− 0.004 [− 0.011–0.003]	− 0.010 [− 0.025–0.005]

The table reports the estimated ATETs for the association between two key household resources and experience of IPV and NPV by women in India under the IPWRA and PSM methods. The figures denote the percentage points reduction in each case. 95% Confidence Intervals are in square brackets next to the point estimates.

**Table 15:** Potential Outcome Means (IPV N = 59,093 and NPV N = 76,580).

POM	Drinking water	Toilet facility
IPV	0.233 [0.225–0.241]	0.229 [0.208–0.249]
NPV	0.049 [0.046–0.053]	0.046 [0.040–0.053]

The table reports the potential outcome means (POMs) for those without access to the key household resources. 95% Confidence Intervals are in square brackets next to the point estimates.

Extrapolating for the country (with around 341 million women in the 15–59 age group in 2016, as per data from https://www.populationpyramid.net/india/2016/), we can project the benefits of providing drinking water access as resulting in reduced IPV for 9.7 million women in India. However, considering the standard error in our point estimates of ATET and POM, the reduction in IPV could range from 2 to 16 million women in the country. We add here the caveat that such extrapolations may not necessarily hold for the entire population, but we nevertheless present them to give an idea of the scale of the potential benefits at a national level. There are certain constraining factors that may limit the effectiveness of WASH facilities, such as high cost of operations and capital maintenance^42,43^.

Coming to availability of water, we observe from Table 14 that, based on the IPWRA analysis, the ATET is 0.006 but not statistically significant. According to this method, water access is not associated with a reduction in IPV. However, as per the PSM results, IPV reduces on an average by 0.035 and the ATET is statistically significant at 1%.

Therefore, availability of toilet facility in a woman’s house has a significant association with reduced violence exercised by their husbands. Some studies have argued that gender roles may not change when key household resources are accessible, e.g. Clancy et al.^44^ state, “Access to modern energy appears to enable women to fulfill their traditional roles (to their satisfaction and wellbeing) rather than bringing significant transformation in gender roles”. However, it has also been argued that if women spend their time savings from access to resources on increasing their income, they may increase their bargaining power within the family^45^.

### IPWRA and PSM results for the relationship between household resources and NPV

Next, with respect to NPV, we find that according to the IPWRA estimates, access to drinking water and toilet facilities are associated with lower NPV by 0.005 and 0.004 respectively (though not statistically significant for toilet access). The corresponding figures from the PSM method are 0.005 and 0.010 respectively. It implies that the lesser the need to step out of the house to access WASH resources, the lower is the exposure to physical violence from non-partners. In Table 15, we observe from the POM estimates that the percentage of women without access to drinking water experiencing NPV is 5%. Applying the estimated ATETs from the IPWRA analysis, we see that access to drinking water can reduce NPV for 371 women in our sample and potentially 1.7 million women in India (which could vary between 0.4 million to 2.8 million women in view of the standard error in the point estimates of ATET and POM).

Finally, we evaluate the robustness of our results to the conditional independence assumption underlying our estimation methods. A concern with both IPWRA and PSM methods is that they do not control for the presence of unobserved covariates that can be correlated with both the treatment and the outcome variables. For example, communities which are more concerned about women’s safety may also have invested more in construction of indoor toilets. The presence of such unobserved factors can bias our estimates of the average treatment effects. If the unobservable characteristics affect the treatment (household resources) and outcome (VAW) variables simultaneously, a ‘hidden bias’ might arise, affecting the robustness of the IPWRA and PSM results. To find out how strongly hidden biases may influence our results, we employ sensitivity analysis following the boundness approach of Rosenbaum^31^. Let Γ be the ratio of the odds of receiving treatment for two matched individuals* i* and *j* with different unobserved characteristics. Following Rosenbaum^28^, we can write:

$$\frac{1}{\Gamma }\le \frac{{P}_{i}/\left(1-{P}_{i}\right)}{{P}_{j}/\left(1-{P}_{j}\right)}\le\Gamma$$

where, P_i_ and P_j_ are the true treatment probabilities that depends on both the observables and the unobservables. Then we can vary the values of Γ starting from 1 and test whether we have overestimated the true treatment effect i.e., whether the estimated treatment effect remains significant across values of Γ^46^. Since our outcome variable is binary, we compute the Mantel–Haenszel test statistic as suggested by Becker and Caliendo^47^ and search for evidence of overestimation of the treatment effects due to the presence of unobservables (see Tables 16 and 17). We find that the assumption of overestimation gets rejected even up to a Γ of 5 which means that, in order to invalidate our results, the unmeasured factor would have to increase the odds of receiving treatment by 5 times compared to an individual without these characteristics. Therefore, we conclude that our IPWRA and PSM results are robust to unobserved confounders.

**Table 16:** Mantel–Haenszel bounds sensitivity analysis for IPV (N = 59,093).

Variables	Γ	MH statistic		Sig. level	
		Overestimation of TE	Underestimation of TE	Overestimation of TE	Underestimation of TE
Drinking water	1	18.332	18.332	0.000	0.000
	1.5	39.489	2.426	0.000	0.008
	2	55.024	17.169	0.000	0.000
	2.5	67.548	28.699	0.000	0.000
	3	78.192	38.220	0.000	0.000
	3.5	87.547	46.364	0.000	0.000
	4	95.961	53.503	0.000	0.000
	4.5	103.653	59.874	0.000	0.000
	5	110.775	65.639	0.000	0.000
Toilet facility	1	34.175	34.175	0.000	0.000
	1.5	56.154	12.866	0.000	0.000
	2	72.460	2.070	0.000	0.019
	2.5	85.699	13.676	0.000	0.000
	3	97.008	23.207	0.000	0.000
	3.5	106.986	31.324	0.000	0.000
	4	115.988	38.416	0.000	0.000
	4.5	124.239	44.728	0.000	0.000
	5	131.895	50.429	0.000	0.000

Γ is the odds of differential assignment due to unobserved factors.

*TE* treatment effects.

**Table 17:** Mantel–Haenszel bounds sensitivity analysis for NPV (N = 76,580).

Variables	Γ	MH statistic		Sig. level	
		Overestimation of TE	Underestimation of TE	Overestimation of TE	Underestimation of TE
Drinking water	1	5.912	5.912	0.000	0.000
	1.5	17.769	5.693	0.000	0.000
	2	26.557	14.050	0.000	0.000
	2.5	33.718	20.695	0.000	0.000
	3	39.859	26.284	0.000	0.000
	3.5	45.297	31.154	0.000	0.000
	4	50.216	35.501	0.000	0.000
	4.5	54.734	39.450	0.000	0.000
	5	58.933	43.084	0.000	0.000
Toilet facility	1	8.105	8.105	0.000	0.000
	1.5	20.226	3.687	0.000	0.000
	2	29.254	12.132	0.000	0.000
	2.5	36.633	18.823	0.000	0.000
	3	42.977	24.437	0.000	0.000
	3.5	48.604	29.318	0.000	0.000
	4	53.701	33.670	0.000	0.000
	4.5	58.388	37.617	0.000	0.000
	5	62.747	41.247	0.000	0.000

Γ is the odds of differential assignment due to unobserved factors.

*TE* treatment effects.

In conclusion, our findings imply that policies and programs aimed at addressing VAW need to recognize the importance of providing key household resources to protect vulnerable women. While WASH facilities are usually provided as part of anti-poverty programs, these resources can have the added benefit of bringing down violence faced by the women in the target households, thereby potentially causing another type of welfare enhancement by improving the well-being of the beneficiaries.

Thus, the findings from our analysis seem to suggest that the Indian government’s recent schemes of building more toilets (Swachh Bharat or Clean India Mission) may produce the additional benefit of reduced violence experienced by vulnerable women. The Indian government has also embarked on a scheme of providing piped water at every rural home within 2024. Our results indicate that such interventions to bring water access to rural households will also contribute to the reduction of violence faced by rural women. Thus, we advocate moving beyond a silo approach in public service delivery and developing citizen centric programmes (instead of isolated interventions), by analysing additional factors such as attitudinal change, cost of provision and feasibility of schemes. There is a clear need for designing more multi-sectoral programming and cross-ministerial coordination. One increasingly popular mechanism for developing cross-sectoral linkages is a ‘one-stop-shop’ or ‘single-window- service’ model, where target beneficiaries of one government service or program receive information, assistance with applications, assessments for and/or direct referrals to other government services or programs^48^. Broadly speaking, there is a need for development policies to be gender sensitive rather than gender blind^49^.

## Supplementary Information

Supplementary Figures.

## Data Availability

The work is based on secondary data that is publicly available at http://rchiips.org/nfhs/factsheet_nfhs-4.shtml. Any requests for the extracts used for this study can be made to the corresponding author.
