# Supplementary material for: The association between access to key household resources and violence against women
Source: Sci Rep. 2023 Jul 28;13:12278. doi: 10.1038/s41598-023-37879-3 (PMC10382593; doi:10.1038/s41598-023-37879-3)
Supplement: Supplementary file 1 — Supplementary Figures. [file 41598_2023_37879_MOESM1_ESM.docx]

**Figure A1: Covariate Balance Graphs-Kernel Density Plots**

**Panel A: IPV**

Electricity

De jure region of residence

De jure place of residence

Education

Ethnicity

Religion

**Panel B: NPV**

Electricity

De jure region of residence

De jure place of residence

Education

Ethnicity

Religion
